# A new era of health leadership

**DOI:** 10.1177/08404704211040817

**Published:** 2021-10-01

**Authors:** Kevin Smith, Megha Bhavsar

**Affiliations:** 1 University Health Network, Toronto, Ontario, Canada.; 2 University Health Network, Toronto, Ontario. Canada.

## Abstract

Traditional models of health leadership are characterized by top-down structures dependent on hierarchy – which emerged historically from military models. With supporting evidence, many of today’s leaders are now working hard to shift their organizations to models of empowered teams and servant leadership with the hopes of inciting a broader cultural shift. The concern is that these early signs of progress could unravel due to the many challenges now exacerbated by COVID-19 and its implications. One such example is fostering respect and civility (i.e. the pillars of empowerment and servant leadership) which is placed at risk during times of change and crisis – more so during a pandemic when command-and-control structures are deemed necessary. The evolution of modern health leadership must be implemented with plans for mitigating related risks. Ultimately, the behaviours that are tolerated during times of stress are what become the value system of any organization.

## Introduction

While it is our profound privilege to serve others in healthcare, it is a calling that places extraordinary demands on its people. Prior to the onset of the COVID-19 pandemic, Canadian healthcare was approaching an extreme collision of several factors: high occupancy rates in hospitals; clinician and staff burnout; equity, diversity and inclusion; continuing financial pressures; physical and digital infrastructure in desperate need of renewal; and beyond. These challenges are only exacerbated by COVID-19.

As essential workers on the frontline of the pandemic, healthcare workers in particular have been working under anxiety-provoking, stressful conditions. Healthcare workers have struggled with the competing demands of caring for their patients, educating learners and conducting research while also ensuring the health and safety of themselves and their families, particularly acute for the healthcare worker population which is approximately 80% of the females in Canada.^1^ Multiple demands continue to test their resilience and capacity, including the threat of new variants; an increased volume of work to meet the pent-up demand for deferred care due to COVID-related restrictions; challenges related to vaccine supply access and uptake; ongoing challenges in daily life related to lockdownsand staff absences; redeployment and changes in staffing models.

While many of today’s leaders have been working hard to shift their organizations to more participative models of leadership with the hopes of inciting a broader cultural shift, it is by no means universal, and the dominant culture remains ‘top down’. The concern is that these early signs of progress in leading environments could unravel due to COVID-19 and its implications and retreat to command-and-control structures.

At the University Health Network (UHN), our renewed focus on a culture of respect and civility, open communication, transparency and compassion is built on our primary value – the needs of patients come first. Complex times call for an accelerated need for a new era of leadership – leadership that counterbalances the necessary command-and-control structures with the reinforcement of the organization’s key values and management of respectful discourse and transparent decision-making. Ultimately, the behaviours that are tolerated especially during times of stress become the value system of any organization.

## Fostering respect and civility to empower teams

Traditional models of health leadership such as command-and-control are characterized by its top-down, hierarchical nature with coercive punishments and an extrinsic reward system.^2,3^ Historically, military operations and crisis management have relied on such top-down, hierarchical approaches. In crisis leadership, there are many actors, significant uncertainty, limited scripts and a great need for improvisation and adaptability as the scale and growth of a novel event outpaces the planning frame, which requires variations in a command-and-control structure.^4^ While these models were ‘accepted’ by Baby Boomers, the following generations have clearly negated their controlled application.

At UHN, we established several command-and-control structures to quickly share information and implement action through our organizational Incident Management System (IMS) while working with colleagues at the provincial IMS table and the Greater Toronto Area IMS tables. Leading through COVID-19 has shown us that there is no ‘one size fits all’ when it comes to leadership models. Leaders must rely on multiple models and styles in order to be successful.^5-7^ Goleman outlines six types of leadership and suggests leaders who have mastered four or more have the best performance.^6^ The four he proposes as particularly important are authoritative, democratic, affiliative and coaching styles, and the most effective leaders can switch between these styles seamlessly as required by the situation.^6^ Even within a specific situation, leaders may adapt their style at different points in order to facilitate progress.^5,6^ This is where emotional intelligence becomes increasingly important. Leaders with high levels of emotional intelligence, including self-awareness and empathy, create environments of information-sharing, trust, healthy risk-taking and learning, whereas leaders with low levels of emotional intelligence create environments of anxiety and fear.^8^ High emotional intelligence is evident in servant leadership which focuses on the needs of others by leading with a high degree of self-awareness and empathy.^7,9,10^ The primary motivation of the servant leader is in service to others and it is from this choice that they aspire to lead.^9,10^ The way we, as leaders, manage our emotions and behaviours is the way we lead, and this will produce a domino effect throughout the organizations we lead. If we desire to lead others towards a sense of purpose even in times of crises, leading with high emotional intelligence must be part of our arsenal. We must model the behaviours we and our teams want. At UHN, we are emphasizing these models by building a foundation of respect and civility.

Incivility can be defined as rude or disruptive behaviour, verbal or non-verbal, failing to act when warranted (withholding information about a patient’s care or refusing to assist a colleague or standing by while others are treated with disrespect); any of which could result in psychological or physiological distress for the people involved.^11^ Most remarkably, incivility is ‘not only what we do, but also what we do not do to intervene when incivility occurs, especially when patient safety is at risk’.^11^ A key component of UHN’s Fostering Respect in the Workplace policy is to model respect and take actions to address incivility. This means speaking up for yourself and perhaps, more importantly, for others. In an increasingly digital age, only accelerated by the pandemic, we must revisit these practices to emphasize digital incivility, including emails, instant messaging and social media.

We know disrespect and incivility can lead to an unpleasant, even hostile, work environment, decreased performance and commitment to the organization, colleagues and patients. There is a documented connection between respectful and civil behaviours and patient safety in healthcare; although much of the research focuses on nursing, the importance of respect and civility across all healthcare professions cannot be underestimated.

Healthcare improvement efforts have focused on the Quadruple Aim to optimize health system performance. Disrespect and incivility directly impact the four elements we, as health leaders, aim to improve: life-threatening errors, preventable complications or harm to a patient^11,12^ are dire and realistic consequences of disrespect and incivility impacting the patient experience; incivility can adversely impact the health and well-being of a provider^13^ and create financial consequences for organizations.^12,14,15^ By virtue of our shared goal to improve health system outcomes, we are incentivized to foster respect and civility where we lead.

## Lead with transparency and open communication to promote psychological safety

Servant leadership requires the opportunity for meaningful input from all team members on most decisions, and in order to do so, staff are provided pertinent information, open communication and a discussion process to facilitate informed decision-making and regular evaluation and feedback mechanisms to course-correct. The desired outcome is an empowered workforce that can challenge all colleagues to work at a higher level, maximizing scope and ultimately leading to an organizational culture that creates people who can build a better tomorrow.

Offering trusted, open and transparent communication to TeamUHN – our employees, medical staff, learners, researchers and volunteers – is an important component of our response. In response to the pandemic and heightened stressors among TeamUHN, we held regular open forums focused exclusively on COVID-19. TeamUHN have the opportunity to submit anonymous questions through Slido, an on-line question and answer tool that helped crowdsource concerns during highly uncertain times. The questions would be addressed by senior leadership based on the number of votes. The sessions were co-hosted by the President & CEO and Vice-President, Public Affairs & Communications. To date, we have hosted over 40 open forums, adjusting frequency based on TeamUHN’s needs – from twice weekly at the pandemic’s peaks, to once biweekly during valleys. There has been an average of 5,000 views and feedback from surveys that indicate staff feel connected, safe and heard. As one respondent described, ‘Open forums provided all your staff and employees with a sense of comfort and stability… it’s like listening to BBC radio during World War II.’

We have set an expectation for respectful discourse and that disrespect and incivility would not be accepted, at times addressing the disrespectful tone of submissions while live. There is little doubt open forums have provided an important avenue for information sharing and organizational improvements; however, the anonymity begs the question – if we are truly a culture of respect and civility, and therefore, safety, does anonymity imply that it is not safe to speak up?

In fact, the literature shows that a dependence on anonymity signals that it is not safe to speak freely, and it can set out a witch hunt for the person who provided the feedback and makes it difficult to address issues, particularly in the context of respect, civility and safety.^16^

In our efforts to lead with trusted, open and transparent communication, and with an eye on continuously improving our culture in line with research, beginning in March 2021, we required TeamUHN to include their names on the submissions to the open forum. Early observations show continued engagement, and although feedback includes some requests for anonymity, we continue to encourage dialogue with the aim of promoting respect, civility and safety. For those who remain unsure of the safety of speaking up, they can contact the Vice-President or CEO confidentially.

## Lead with compassion to tackle wicked problems

In this new era of leadership, we must acknowledge the privilege of the positions we hold and confront the systemic issues. COVID-19 has exposed many inequities in our society, none of which can or should be delayed to a post-pandemic time. In 2020, we were confronted with violent racist acts in North America, shedding light on the truth that racism exists everywhere, including Canadian healthcare. As health leaders, we have a moral obligation to stand in solidarity with Black, Indigenous and People of Colour (BIPOC) and denounce anti-Indigenous and anti-Black racism and all racism. We must also have the humility to acknowledge what we do not know and the willingness to learn from those who have lived experience. At UHN, we have developed our Anti-Black Racism and Anti-Racism Policy, and are developing an Anti-Racism and Anti-Black Racism Strategy. The shocking discoveries at residential schools have strengthened our commitment to engage with Indigenous persons and address the inherent bias in our system in true partnership. It cannot be underscored that this work is not possible without the leadership and true partnership of BIPOC individuals and groups.

We also commit to delivering the highest quality care for our underserved patient populations. We know that racialized populations and low-income populations are at higher risk of COVID-19. In fact, in the city of Toronto, racialized people comprised more COVID-19 cases (77%) than non-racialized people and made up more of the cases that were hospitalized due to COVID-19 (78% after age standardization).^17^ Similarly, lower-income households comprised about 48% of COVID-19 cases, and 56% of people who were hospitalized due to COVID-19 came from lower-income households, after age standardization.^17^ A new era of leadership in healthcare must consider the needs of these populations and the actions we, as leaders, take to serve often marginalized communities.

Race and racism are closely tied to health outcomes, and as health leaders, if we are truly committed to fostering respect and civility and empowering our people, we must actively dismantle institutional racism by identifying, preventing and removing barriers in delivering care, employment, education and research.

## Advancing the way we lead for future leaders

The world is changing rapidly. The emergence of COVID-19 led the rapid and changing integration of knowledge, training and experience across healthcare.^18^ COVID-19 has shown us that healthcare has the possibility to adapt more quickly than imagined. As health leaders, we adapted to the needs of combatting the virus – from physical distancing, masking and remote working where possible to leaps in virtual care and other technology-enabled advances.

It was estimated that the doubling time of medical knowledge was 50 years in 1950; by 1980, it was 7 years, and it was projected to be just over two months in 2020.^19^ Healthcare environments are becoming highly complex and uncertain as COVID-19 has demonstrated – the next generation of healthcare providers must be trained for these environments while knowing that what is relevant for our generation may very well be irrelevant for the next. This is where critical thinking, knowledge sharing and leveraging data will be important as healthcare embraces digital tools and Big Data. As today’s educators and mentors, we have a responsibility to instill this mentality of critical thinking into future providers, and this will ultimately mean we must embrace diversity and dissent as part of the decision-making process. It is also up to us to seek the perspectives of these generations as they are digital experts.

With the increased use of technology in healthcare, digital respect and civility will need to be a core emphasis in building an empowered organizational culture. It will be up to leaders to set these norms for behaviour and create the effect we wish to see.

Most importantly, we must not forget the human side of leadership when ushering in this technological age. Compassion is at the heart of healthcare. Hodges, Paech and Bennett describe a healthcare system in which the best leaders and technologies collaborate to anchor care in compassion – moving away from a dichotomy of humans vs machine.^20^ As health leaders, we need to provide people with the tools they need to their job – the right technologies for patients and providers – in preparation of future healthcare providers and leaders.

## Conclusion

As Canada’s leading academic health sciences centre, UHN is committed to a new era of leadership – one that emphasizes our values of transparency and open communication, compassion, and respect and civility – especially in the most challenging of times. In both a pandemic and a world in which COVID-19 has been accommodated as part of our health system, how and where we deliver care, research and education is rapidly changing with more digital tools at our hands. As COVID-19 required command-and-control structures give way, the groundwork is there for us as health leaders to embrace a new era of leadership, to develop an era of a diverse and empowered workforce and a culture that aligns with the values we want to instill. Leading in times of crisis is an opportunity to build the best culture for our providers, researchers, educators and patients.
